# Prevalence and predictive factors of osteoporosis in Thai systemic sclerosis

**DOI:** 10.1038/s41598-021-88792-6

**Published:** 2021-05-03

**Authors:** Wiriya Chuealee, Chingching Foocharoen, Ajanee Mahakkanukrauh, Siraphop Suwannaroj, Chatlert Pongchaiyakul, Ratanavadee Nanagara

**Affiliations:** Department of Medicine, Faculty of Medicine, Khon Kaen University, Khon Kaen, 40002 Thailand

**Keywords:** Systemic sclerosis, Osteoporosis

## Abstract

Systemic sclerosis (SSc) is recognized as a chronic inflammatory disease and several SSc-associated factors may increase the risk of osteoporosis and its related fractures. To determine the prevalence and predictive factors of osteoporosis in Thai SSc, a cross-sectional study was designed in adult SSc patients at Scleroderma clinic, Khon Kaen University Hospital. The prevalence of osteoporosis with the 95% confidence interval (CI) were determined and the odds ratio (OR) with 95%CI were assessed the clinical association with osteoporosis. A total of 205 SSc patients were recruited with the female to male ratio of 2.7:1. The majority of cases were diffuse SSc subset (83.4%) with a disease duration < 5 years (62.9%). The overall prevalence of osteoporosis was 29.3% (95%CI 23.1–36.0). After an age adjusted analysis, the respective prevalence of osteoporosis at lumbar spine (LS) in women and men was 26.3% and 10%, while the prevalence of osteoporosis at the femoral neck (FN) in women and men was 11% and 2.1%. Low BMI (≤ 18.5 kg/m^2^) and menopause were associated with osteoporosis at both the LS and FN. Using multivariate analysis, low BMI and menopause were associated with osteoporosis at LS (OR 7.78 and 5.32, respectively), while low BMI was also associated with osteoporosis at LS in pre-menopausal women. In conclusion, the prevalence of osteoporosis in Thai SSc was 29.3%. Osteoporosis at the LS is more common than FN in both men and women. Low BMI was associated with osteoporosis in overall SSc and pre-menopausal women, while only menopause was associated with osteoporosis at the FN.

## Introduction

Osteoporosis is a skeletal disease characterized by low bone mass and microarchitecture deterioration of bone tissue with a consequent rise in fragility fractures^1^. Osteoporosis has increasingly become a global concern because it is age-related and has an exponentially-increased incidence, morbidity, mortality and expense^2–4^.

Systemic sclerosis (SSc), a rare rheumatic disease, is characterized by progressive skin fibrosis, vasculopathy, and internal organ involvement^5^. While the pathophysiology of the disease is unclear^6^ and limited evidences on the treatment for some complications of SSc^7^, SSc is typically progressive, resulting in death, disability, and decreased quality of life^8–10^. It is well known that the extent of skin and internal organ involvement are main features in SSc patients; however, SSc has been recognized as another potential chronic inflammatory disease and several factors related with SSc can affect bone tissue, including immobilization (due to contracture and joint involvement), malabsorption that causes malnutrition, premature menopause, renal insufficiency, and glucocorticoid use^11,12^. Therefore, patients with SSc may have an increased risk of osteoporosis and its associated fractures.

The prevalence and associated factors of osteoporosis in SSc patients have been documented in previous epidemiologic studies; however, findings differ among studies. These contradictory findings are due to several factors, including limited sample size, study design, duration and severity of disease, proportion of different SSc subsets, and menopausal status^12–19^. Due to the lack of epidemiological evidence on osteoporosis and associated factors with osteoporosis in Thai SSc patients, we therefore aimed to determine the prevalence of osteoporosis in Thai SSc patients and its associated factors.

## Patient and methods

A cross-sectional study was conducted in adult patients (over 15 years of age) with SSc who attended the Scleroderma Clinic at Srinagarind Hospital, Khon Kaen University, Thailand, from November 2012 to August 2013. All patients met the requirements for diagnosis of SSc based on the criteria of American College of Rheumatology^20^. Cases were categorized as lcSSc (limited cutaneous SSc) or dcSSc subset as defined by LeRoy et al.^21^. We excluded the patients (a) who were diagnosed with overlap with other connective tissue diseases, cancer undergoing chemotherapy, chronic kidney disease, and hypothyroidism, (b) who were pregnant or breast feeding, and (c) who had been evaluated for bone mineral density (BMD) or nuclear medicine within 3 months.

Based on a previous estimate of the prevalence of osteoporosis in SSc (19.4%)^12^ with a sampling variability of 5%, it was estimated that a sample size of at least 241 was needed for statistical adequacy to estimate the true prevalence of osteoporosis. After the retention period and a preliminary analysis, we found the prevalence of osteoporosis was 58 of 205 cases (29.3%) (95% confidence interval (CI) 23.1–36.0). We thus recalculated the sample size and collected 205 instead of 241 cases in order to achieve better patient care and reduce the cost of the study. This study was approved by the Human Research Ethics Committee of Khon Kaen University (HE551280). All eligible patients signed informed consent before enrolling in the study.

Demographic and clinical characteristics including age, sex, menopausal status, duration of disease, SSc subset, and family history of osteoporosis and fracture were collected. BMD at the lumber spine (LS) and femoral neck (FN) were measured using dual energy X-ray absorptiometry (DXA) by GE Lunar DPX Duo Densitometer. Osteoporosis was defined by a T-score of -2.5 SD, compared with the peak young adult mean for Thai women. The 25-hydroxy vitamin D [25(OH)D] level using direct competitive chemiluminescence immunoassay, thyroid and thyroid stimulating hormones, parathyroid hormone, calcium and phosphorus levels, and inflammatory markers [erythrocyte sedimentation rate (ESR) and C-reactive protein (CRP)] were measured.

In this study, duration of disease was the interval between the date of osteoporosis assessment and the first SSc symptoms. Pulmonary fibrosis was defined when interstitial fibrosis was detected by either chest radiography or high resolution computed tomography (HRCT). Pulmonary arterial hypertension was defined when mean pulmonary arterial pressure was ≥ 25 mmHg as confirmed by right cardiac catheterization. Gastrointestinal involvement of SSc included any gastrointestinal symptoms (i.e., malabsorption, constipation, ileus, or pseudo-intestinal obstruction). Vitamin D insufficiency and deficiency was defined if 25(OH)D levels were < 30 and < 20 ng/ml, respectively.

### Statistical analysis

Demographic data are categorized and summarized using descriptive statistics. Categorical data were presented as proportions or percentages. Continuous data were presented as means with standard deviations (SD) or medians with interquartile ranges (IQR) as appropriate. The overall prevalence of osteoporosis, the prevalence of osteoporosis at the LS and FN with their 95% confidence interval (CI) were calculated. The odds ratio with 95%CI and p-value were used to determine the association between clinical characteristics and osteoporosis at the LS and FN. Continuous data were analyzed using the student *t*-test or Mann Whitney U-test as appropriate. The variables with a *p* value < 0.20 were entered into a multiple logistic regression model. The backward elimination method was applied for model fitting. Variables were tested for significance using the Wald X^2^ statistic: all statistical tests were two-tailed. *p* values < 0.05 were considered to be statistically significant. Data analysis was performed using version 11.2 of STATA (StataCorp., College Station, TX, USA).

The study was designed by the authors and approved by the Human Research Ethics Committee of Khon Kaen University as per the Helsinki Declaration and the Good Clinical Practice Guidelines (HE551280). All eligible patients signed informed consent before enrollment. The sponsor had no role in the study.

## Results

A total of 205 cases were recruited for the final analysis. There were 149 women and 56 men with a ratio of 2.7 to 1. The mean age and BMI at the time of study were 53.6 ± 10.6 years (range, 27–81) and 21.1 ± 4.0 kg/m^2^ (6.7–31.4), respectively. The majority (62.9%) had a duration of disease < 5 years and most (83.4%) were the dcSSc subset. Approximately one-fifth of the cases (n = 44) had a modified Rodnan skin score (mRSS) of more than 20 points.

The overall median 25(OH)D level for all patients was 31.5 ng/ml (IQR 25.2–37.1). Vitamin D insufficiency and deficiency were 29.3% and 13.2%, respectively. Women had a significantly lower vitamin D level, and a higher rate of vitamin D insufficiency and deficiency than men (*p* < 0.001, *p* = 0.004, *p* = 0.006, respectively) (Table 1).

**Table 1:** Clinical characteristics classified by sex.

Factor	Overall N = 205	Women N = 143	Men N = 62	*p* value between female and male
Age at study (years); mean ± SD (range)	53.6 ± 10.6 (27–81)	52.8 ± 11.5 (27–81)	55.7 ± 8.3 (39–77)	0.12
BMI (kg/m^2^); mean ± SD (range)	21.1 ± 4.0 (6.7–34.0)	21.3 ± 4.5 (6.7–34)	20.7 ± 2.6 (14.4–25.6)	0.32
Duration of disease > 5 years (%)	76 (37.1)	55 (38.5)	21 (33.9)	0.53
mRSS > 20 (%)	42 (20.5)	25 (17.5)	17 (27.4)	0.11
Median 25(OH)D level; median (IQR)	31.5 (25.2–37.1)	30.2 (22.4–35.2)	34.9 (28.8–43.9)	< 0.001*
Vitamin D deficiency (%)	27 (13.2)	25 (17.5)	2 (3.2)	0.006*
Vitamin D insufficiency (%)	60 (29.3)	45 (31.5)	15 (24.2)	0.004*
**BMD**				
L1–4				
BMD (g/cm^2^); mean ± SD (range)	0.79 ± 0.13 (0.29–1.14)	0.77 ± 0.13 (0.29–1.14	0.87 ± 0.11 (0.57–1.14)	0.001*
T-score; median (IQR)	− 1.0 (− 1.6, − 0.3)	− 1.2 (− 1.8, − 0.6)	− 0.45 (− 1.0, − 0.1)	< 0.001*
Z-score; median (IQR)	− 0.2 (− 0.9, 0.5)	− 0.4 (− 1.1, 0.3)	0.35 (− 0.3, 0.7)	< 0.001*
Osteopenia (%)	92 (44.9)	66 (46.2)	26 (41.9)	
Osteoporosis (%)	58 (28.3)	46 (32.2)	12 (19.4)	0.07
Femoral neck				
BMD; median (IQR)	0.74 (0.65–0.80)	0.70 (0.63–0.77)	0.79 (0.72–0.85)	< 0.001*
T-score; median (IQR)	− 1.3 (− 1.9, − 0.7)	− 1.5 (− 2.1, − 0.9)	− 1.05 (− 1.6, − 0.4)	< 0.001*
Z-score; median (IQR)	− 0.3 (− 0.9, 0.4)	− 0.4 (− 1.0, 0.4)	0.2 (− 0.4, 0.5)	0.04*
Osteopenia (%)	118 (57.6)	90 (62.9)	28 (45.2)	
Osteoporosis (%)	18 (8.8)	16 (11.2)	2 (3.2)	0.06

*BMI* Bone mass index, *mRSS* Modified Rodnan skin score, *BMD* Bone mineral density, *L1–4* Lumbar spine level 1–4.

*Statistical significant.

The overall prevalence of osteoporosis was 29.3% (95%CI 23.1–36.0). The prevalence of osteoporosis at the LS in SSc patients was 28.3% (95% CI 22.2–35.0), and it was significantly higher in women than in men (32.2% vs. 19.4%) (Table 1). While, the prevalence of osteoporosis at FN was lower (8.8%, 95% CI 5.3–13.5) and trend to be higher in women than in men without significant difference (11.2% vs. 3.2%, *p* = 0.06). The prevalence of osteoporosis at both sites (LS and FN) was 7.8% (95%CI 4.5–12.4) and there was no significantly difference between women and men (6.8% vs 1.0%, *p* = 0.16). In this study, the age-adjusted prevalence of osteoporosis in women and men was 26.3% and 10% at LS and 11.0% and 2.1% at FN. The clinical characteristics including the vitamin D level and BMD, classified by sex, were shown in Table 1.

The overall median T-score BMD at LS and FN in dcSSc patients was − 1.8 (IQR − 2.6, − 1.0) and − 1.4 (IQR − 1.9, − 0.7), respectively. The overall median T-score BMD at LS and FN in lcSSc patients was − 1.9 (IQR − 2.8, − 1.0) and − 1.3 (IQR − 1.6, − 0.4), respectively. The BMD of both LS and FN were not different between dcSSc and lcSSc (*p* = 0.82 and 0.25, respectively), as well as the prevalence of osteoporosis at both LS and FN, which was comparable between dcSSc and lcSSc (28.1% vs 27.2%, *p* = 0.93 at LS and 7.6% vs 6.1%, *p* = 0.99 at FN).

In this study, low BMI (≤ 18.5 kg/m^2^) and menopause were associated with osteoporosis at both LS and FN. However, other factors including SSc subset, severity of skin tightness, glucocorticoid used, vitamin D insufficiency or deficiency, smoking history, previous fracture and family history of fracture were not associated with osteoporosis at any sites (Tables 2 and 3). In multivariate analysis, low BMI and menopause increased risk of osteoporosis at the LS [OR 7.78 (95%CI 3.21–18.88) and 5.32 (95%CI 1.84–15.35), respectively], while only low BMI was significantly associated with osteoporosis at FN [OR 4.54 (95%CI 1.41–13.64)].

**Table 2:** Clinical factors associated with osteoporosis at the lumbar spine.

Factor	No osteoporosis N = 147	Osteoporosis N = 58	Odds ratio (95%CI)
Age (years); mean ± SD	51.4 ± 9.6	59.1 ± 11.3	< 0.001*
Age > 50 years (%)	80 (54.2)	46 (79.3)	3.21 (1.51–7.18)
Sex Men (%)	44 (78.6)	12 (21.4)	0.61 (0.30–1.26)
Women (%)	103 (69.1)	46 (30.9)	
Men ≥ 70 years (%)	0	3 (5.2)	NA
Menopause (%)	60 of 97 (61.9)	40 of 46 (87.0)	4.11 (1.51–12.91)*
dcSSc subset (%)	123 (71.9)	48 (28.1)	1.04 (0.45–2.40)
Duration of disease ≥ 5 years (%)	54 (36.7)	22 (37.9)	1.04 (0.66–1.62)
Anti-topoisomerase I positive (%)	112 of 147 (76.7)	48 of 58 (82.8)	1.32 (0.73–2.39)
Anti-centromere positive (%)	6 of 14(6.2)	5 of 14(8.6)	1.28 (0.61–2.68)
mRSS > 20 (%)	28 (19.1)	14 (24.1)	1.35 (0.60–2.94)
BMI ≤ 18.5 kg/m^2^ (%)	21 (14.3)	30 (51.7)	3.24 (2.16–4.86)*
Internal organ involvement			
Gastrointestinal involvement (%)	45 (30.6)	17 (29.3)	0.94 (0.45–1.91)
Pulmonary fibrosis (%)	102 (69.4)	41 (70.7)	1.06 (0.52–2.22)
Pulmonary arterial hypertension (%)	14 (9.5)	2 (3.5)	0.34 (0.04–1.56)
Inflammatory marker			
CRP > 5 mg/dl (%)	53 (36.1)	23 (40.4)	1.14 (0.73–1.78)
ESR > 20 mm/hr (%)	121 (82.3)	53 (91.4)	1.89 (0.82–4.35)
PTH > 65 pg/ml (%)	21 (14.3)	6 (10.3)	0.76 (0.36–1.60)
Vitamin D deficiency (%)	19 (12.9)	8 (13.8)	1.05 (0.56–1.97)
Treatment			
Current prednisolone used > 10 mg/d (%)	1 (1.4)	2 (3.5)	1.79 (0.66–4.90)
Current immunosuppressant used (%)	71 (48.3)	30 (51.7)	1.10 (0.71–1.71)
Current PPI used (%)	133 (90.5)	57 (98.3)	4.5 (0.67–30.3)
Current smoking (%)	9 (6.1)	1 (1.7)	0.34 (0.05–2.22)
Current coffee drinking (%)	42 (28.6)	10 (17.2)	0.61 (0.33–1.12)
Previous history of fracture (%)	11 (7.5)	5 (8.6)	1.11 (0.52–2.39)
Family history of osteoporosis fracture (%)	5 (3.4)	2 (3.5)	1.01 (0.31–3.33)

*CI* Confidence interval, *dcSSc* Diffuse cutaneous systemic sclerosis, *mRSS* Modified Rodnan skin score, *CRP* C-reactive protein, *ESR* Erythrocyte sedimentation rate, *PTH* Parathyroid hormone, *PPI* Proton pump inhibitor.

*Statistical significant.

**Table 3:** Clinical factors associated with osteoporosis at the femoral neck.

Factor	No osteoporosis N = 187	Osteoporosis N = 18	Odds ratio (95%CI)
Age (years); mean ± SD	52.9 ± 10.3	60.8 ± 12.1	0.002*
Age > 50 years (%)	112 (59.9)	14 (77.8)	2.34 (0.70–10.12)
Sex Men (%)	55 (98.2)	1 (1.8)	0.14 (0.02–10.9)
Women (%)	132 (88.6)	17 (11.4)	1
Men ≥ 70 years (%)	2 (1.1)	1 (5.6)	5.44 (0.08–107.92)
Menopause (%)	85 of 127 (66.9)	15 of 16 (93.8)	7.41 (1.06–319.27)*
dcSSc subset (%)	157 (91.8)	14 (8.2)	0.89 (0.24–3.29)
Duration of disease ≥ 5 years (%)	69 (36.9)	7 (38.9)	1.08 (0.44–2.67)
Anti-topoisomerase I positive (%)	143 of 187 (76.9)	17 of 18 (94.4)	4.68 (0.64–34.17)
Anti-centromere positive (%)	13 of 14 (7.0)	1 of 14 (5.6)	0.80 (0.11–5.57)
mRSS > 20 (%)	37 (19.8)	5 (27.8)	1.56 (0.41–5.03)
BMI ≤ 18.5 kg/m^2^ (%)	40 (21.4)	11 (61.1)	4.75 (1.94–11.6)*
Internal organ involvement			
Gastrointestinal involvement (%)	56 (30.0)	6 (33.3)	1.17 (0.34–3.57)
Pulmonary fibrosis (%)	129 (69.0)	14 (77.8)	1.57 (0.47–6.84)
Pulmonary arterial hypertension (%)	16 (8.6)	0	NA
Inflammatory marker			
CRP > 5 mg/dl (%)	71 (38.0)	5 (29.4)	0.70 (0.26–1.92)
ESR > 20 mm/h (%)	157 (84.0)	17 (94.4)	3.03 (0.42–21.94)
PTH > 65 pg/ml (%)	23 (12.3)	4 (22.2)	1.88 (0.67–5.30)
Vitamin D deficiency (%)	23 (12.3)	4 (22.2)	1.88 (0.67–5.30)
Treatment			
Current steroid > 10 mg/d (%)	4 (2.1)	0	NA
Current immunosuppressant using (%)	92 (49.2)	9 (50.0)	1.03 (0.43–2.49)
Current PPI used (%)	173 (92.5)	17 (94.4)	1.34 (0.19–9.41)
Current smoking (%)	10 (5.4)	0	NA
Current coffee drinking (%)	50 (26.7)	2 (11.1)	0.37 (0.09–1.55)
Previous history of fracture (%)	15 (8.0)	1 (5.6)	0.69 (0.10–4.89)
Family history of osteoporosis fracture (%)	7 (3.7)	0	NA

*CI* Confidence interval, *dcSSc* Diffuse cutaneous systemic sclerosis, *mRSS* Modified Rodnan skin score, *CRP* C-reactive protein, *ESR* Erythrocyte sedimentation rate, *PTH* Parathyroid hormone, *PPI* Proton pump inhibitor, *NA* Not applicable.

*Statistical significant.

A further subgroup analysis in pre-menopausal women with low BMI was performed. The prevalence of osteoporosis at LS and FN was 13.9% (95%CI 3.2–24.7) and 2.3% (95%CI 0.3–7.0), respectively. We found that only BMI ≤ 18.5 kg/m^2^ was associated with osteoporosis at LS but not FN. And there was no association between other clinical parameters and osteoporosis in pre-menopausal SSc women.

## Discussion

In the present study, the overall prevalence of osteoporosis in our Thai SSc patients was 29.3%, which is higher than previous reports from Taiwan and China (17–19%)^12,18^ and some Caucasian populations (3.3%)^14^ but less than Brazilians and Moroccans reports (31.7–33%)^15,22^. The high prevalence of osteoporosis in Thais, Brazilians, and Moroccans might be explained by the higher proportion of the dcSSc subset compared to more prevalent lcSSc subset reported by Western countries. A previous study demonstrated a lower BMD in dcSSc than in lcSSc and intermediate SSc; probably because of extensive skin tightness and early internal organ involvement. The high disease activity might be related to limited daily activities which is a well-known risk factor for osteoporosis in the general population^14^. In contrast to our finding, the BMD at both LS and FN were comparable between dcSSc and lcSSc. Moreover, dcSSc subset was not associated with increased risk of osteoporosis in Thai SSc patients. This finding was due to a limited number of lcSSc in our study. The prevalence and associated factors for osteoporosis in SSc in the literature and our study are presented in Table 4.

**Table 4:** Comparison of prevalence and associated factors for osteoporosis in SSc patients.

Study	This study	Yuen et al.^12^	Mok et al.^18^	Sampaio-Barros et al.^16^	Ibn Yacoub et al.^22^	Frediani et al.^14^	Souza et al.^15^	Neumann et al.^32^
Year	2013	2008	2012	2000	2012	2004	2006	2000
Number of patients	205	159	84	74	60	47	43	30
Ethnic	Thai	Taiwan	Chinese	89% Caucasian	Moroccan	Whites	Brazilians	93% Caucasian
Female to male ratio	2.7:1	4.5:1	8:1	100% female	–	100% female	–	100% female
dcSSc to lcSSc ratio	1.5:1	–	1:3.8	1:1.9	3.8:1	1:1.6	–	1.7:1
Disease duration (years)	83.4% (≥ 5 years)	11.5	7.8	10.7	9.6	–	13.2	9.5
Proportion of postmenopausal women (%)	69.6	–	–	45.9	–	57	100	80
Prevalence of OP at lumbar spine (%)	28.3	18.7	17.0	23	33	30	32.5	3.3
Prevalence of OP at femoral neck (%)	8.8	–	6.0	–	51	–	51.1	6.7
Factors association with BMD								
BMD of lumbar spine	BMI < 18.5 kg/m^2^ (OR 7.78) Menopause (OR 5.32)	–	Age (coefficient − 0.34; * p* = 0.03) Menopause (coefficient − 0.31; * p* = 0.04)	BMI (R2 22.5; * p* = 0.001)	–	Internal organ involvement	Weight (R2 0.44; * p* = 0.01) Lean mass (R2 0.40; * p* < 0.001) SSc (R2 0.47; * p* = 0.04)	–
BMD of femoral neck	BMI < 18.5 kg/m^2^ (OR 4.54)	–	Age (coefficient − 0.50; * p* = 0.001) BMI (coefficient 0.24; * p* = 0.02)	BMI (R2 35.3; * p* = 0.001)	–	Internal organ involvement	Duration of menopause (R2 0.38; * p* < 0.01) Lean mass (R2 0.30; * p* < 0.001) SSc (R2 0.42; * p* = 0.03)	–
BMD of at least 1 skeletal site	–	Older age	–	–	Disease duration (R2 0.47; * p* < 0.01) Visual analogue scale of pain (R2 0.14; * p* < 0.05) Erosive arthropathy (R2 0.77; * p* < 0.01) Malabsorption syndrome (R2 1.07; * p* < 0.001) Anti-Scl70 positive (R2 0.65; *p* < 0.01)	dcSSc BMI Older age Years since menopause	–	–

*OP* Osteoporosis, *dcSSc* Diffuse cutaneous systemic sclerosis, *lcSSc* Limited cutaneous systemic sclerosis, *BMI* Body mass index, – No data, *BMD* Bone mineral density.

We found that a low BMI (BMI ≤ 18.5 kg/m^2^) is increased risk of osteoporosis in Thai SSc patients including pre-menopausal women and men. This finding is consistent as in general population^22^. Despite low BMI is a strong predictor of osteoporosis in our study, the exact mechanism for the development of osteoporosis has not been elucidated. Patients with SSc are prone to having low BMI due to decreased dietary intake, esophageal hypomotility, and intestinal malabsorption^15,16,23^. It has been accepted that low BMI is a symbolic clinical sign of malnutrition^23^. Around one-third of the SSc patients are at risk of malnutrition particularly in the patients with shorter duration of disease, more disease severity and having gastrointestinal involvement^24^. Moreover, lean mass and fat mass are decreased in SSc patients, which contributes to a low BMI^18,25^. Loss of lean mass in patients with SSc is related to physical inactivity due to joint contracture, arthritis, muscle wasting, and malnutrition, while low fat mass is associated with a low estrogen level^26^. Previous research demonstrated that low BMI is related to a low level of insulin-like growth factor (IGF-1) and low estrogen levels^27^; both of which are well-known associated factors with osteoporosis. Therefore, the SSc patients who are at risk of malnutrition or low BMI even in premenopausal women should be screened for early identification, closed monitoring, awareness of the complication and giving the proper management for osteoporosis.

Our study did not demonstrate the associations among intestinal involvement, vitamin D deficiency, and osteoporosis; all of which may be the result of the malabsorption problem occasionally found in the dcSSc subgroup of SSc. However, our unpublished data demonstrated that several physical limitations were associated with malnutrition status in Thai SSc (viz., daily activity, self-feeding capability, hand grip ability, painful digital ulcers, and narrow mouth aperture). Physical limitations and self-feeding capability might thus increase the risk of malnutrition, leading to osteoporosis in Thai SSc patients. The results from current study provides clinical clues that may help to evaluate osteoporosis in SSc patients. Further research is needed to determine whether improvement of nutritional status at disease onset can decrease the prevalence of osteoporosis in SSc patients.

We did not found the associations between disease activities and osteoporosis risk in the present study including severe skin tightness, dcSSc subset, internal organ involvement, or inflammatory markers (ESR, CRP). The findings in our study are inconsistent with a case–control study which found that digital ulcers, presence of anti-centromere antibody, low lean mass and high number of previous fracture were an independent risks of low trabecular volumetric bone mineral density at tibia^28^. The authors also proposed the possible role of vasculopathy on the bone demineralization process^28^. The dissimilar findings with ours might be explained by the different method and different site of bone mineral density testing. According to the pathogenesis of SSc, the inflammatory process was not remarkable, thus the inflammatory marker was not associated with osteoporosis in our patients unlike other rheumatic diseases (i.e., rheumatoid arthritis)^29^.

Not only BMD assessment but also bone quality assessment has been evaluated in SSc. Poor bone quality evaluated by trabecular bone score (TBS) was reported among SSc patients. The prevalence of low TBS seemed to be more frequently found in SSc than in other connective tissue disease^30^. Moreover, there was a negative correlation between TBS and Dickkopf-1 serum levels which was a natural inhibitor of the Wnt signaling pathway promoting osteoclastogenesis^31^. According to the poor bone quality detection among SSc, therefore both of bone mass and bone quality should be cooperatively evaluated and further study of bone quality and the risk of fracture among SSc patients is suggested.

It has been accepted that treatment options for oral bisphosphonates may be limited due to esophageal disease, whereas treatment options for raloxifene and hormone therapy may be limited due to potential thrombotic complications. To avoid the gastrointestinal tract, intravenous zoledronate, denosumab, and teriparatide are better options for treating osteoporosis in SSc patients. However, the treatment option of osteoporosis was not our objective of the study, so we did not evaluate the treatment of osteoporosis in our SSc patients. The further research is needed to assess the outcome.

There are a number of limitations to the present findings. First, we did not perform tests for gastrointestinal malabsorption in severely malnourished SSc patients even though such a condition can be related to development of osteoporosis. Secondly, we did not check the hormonal status in pre-menopausal women even though low estrogen is associated with early development of osteoporosis. Third, we did not use a food intake questionnaire for evaluating nutritional status. Fouth, we did not exclude low BMI. However, this study was conducted in a single center with a large number of SSc patients. The current study included several important parameters, including vitamin D level, parathyroid hormone, thyroid and thyroid stimulating hormones, and inflammatory markers, which might associate with development of osteoporosis in SSc patients and also excluded the comorbid conditions that might confound BMD measurement (i.e., cancer undergoing chemotherapy, chronic kidney disease, hypothyroidism). Moreover, the findings from this study, including the prevalence and factors associated with osteoporosis in SSc patients, can be used for developing treatment guidelines to prevent osteoporosis.

In conclusion, the overall prevalence of osteoporosis in Thai patients with SSc was 29.3%, with women having a higher prevalence than men. Osteoporosis of the lumbar spine is more common than osteoporosis of the femoral neck in both gender. Low BMI (≤ 18.5 kg/m^2^) and menopausal status are associated with osteoporosis at the lumbar spine and femoral neck.

## Data Availability

Data and material available upon request.

## References

[1] NIH Consensus Development Panel on Osteoporosis Prevention, Diagnosis, and Therapy. Osteoporosis prevention, diagnosis, and therapy. *JAMA* **285**, 785–795 (2001).

[2] Cooper C (1997). The crippling consequences of fractures and their impact on quality of life. Am. J. Med..

[3] Cosman F (2014). Clinician’s guide to prevention and treatment of osteoporosis. Osteoporos. Int..

[4] Cooper C, Campion G, Melton LJ (1992). Hip fractures in the elderly: a world-wide projection. Osteoporos. Int..

[5] Gabrielli A, Avvedimento EV, Krieg T (2009). Scleroderma. N. Engl. J. Med..

[6] Varga J, Trojanowska M, Kuwana M (2017). Pathogenesis of systemic sclerosis: recent insights of molecular and cellular mechanisms and therapeutic opportunities. J. Scleroderma Relat. Disord..

[7] Smith V (2018). Systemic sclerosis: state of the art on clinical practice guidelines. RMD Open.

[8] Hissaria P (2011). Survival in scleroderma: results from the population-based South Australian Register. Intern. Med. J..

[9] Simeón CP (2003). Mortality and prognostic factors in Spanish patients with systemic sclerosis. Rheumatology (Oxford).

[10] Al-Dhaher FF, Pope JE, Ouimet JM (2010). Determinants of morbidity and mortality of systemic sclerosis in Canada. Semin. Arthritis Rheum..

[11] Loucks J, Pope JE (2005). Osteoporosis in scleroderma. Semin. Arthritis Rheum..

[12] Yuen SY, Rochwerg B, Ouimet J, Pope JE (2008). Patients with scleroderma may have increased risk of osteoporosis. A comparison to rheumatoid arthritis and noninflammatory musculoskeletal conditions. J. Rheumatol..

[13] La Montagna G, Vatti M, Valentini G, Tirri G (1991). Osteopenia in systemic sclerosis. Evidence of a participating role of earlier menopause. Clin. Rheumatol..

[14] Frediani B (2004). Bone mineral density in patients with systemic sclerosis. Ann. Rheum. Dis..

[15] Souza RBC, Borges CTL, Takayama L, Aldrighi JM, Pereira RMR (2006). Systemic sclerosis and bone loss: the role of the disease and body composition. Scand. J. Rheumatol..

[16] Sampaio-Barros PD (2005). Prognostic factors of low bone mineral density in systemic sclerosis. Clin. Exp. Rheumatol..

[17] Carbone L, Tylavsky F, Wan J, McKown K, Cheng S (1999). Bone mineral density in scleroderma. Rheumatology (Oxford).

[18] Mok CC, Chan PT, Chan KL, Ma KM (2013). Prevalence and risk factors of low bone mineral density in Chinese patients with systemic sclerosis: a case-control study. Rheumatology (Oxford).

[19] Avouac J (2012). Increased risk of osteoporosis and fracture in women with systemic sclerosis: a comparative study with rheumatoid arthritis. Arthritis Care Res. (Hoboken).

[20] Preliminary criteria for the classification of systemic sclerosis (scleroderma). Subcommittee for scleroderma criteria of the American Rheumatism Association Diagnostic and Therapeutic Criteria Committee. *Arthritis Rheum* **23**, 581–590 (1980). 10.1002/art.1780230510 7378088

[21] LeRoy EC (1988). Scleroderma (systemic sclerosis): classification, subsets and pathogenesis. J. Rheumatol..

[22] Ibn Yacoub Y (2012). Bone density in Moroccan women with systemic scleroderma and its relationships with disease-related parameters and vitamin D status. Rheumatol. Int..

[23] Maeda, K., Ishida, Y., Nonogaki, T. & Mori, N. Reference body mass index values and the prevalence of malnutrition according to the Global Leadership Initiative on Malnutrition criteria. *Clinical nutrition (Edinburgh, Scotland).* Clin Nutr. **39**, 180–184 (2020) 10.1016/j.clnu.2019.01.011 30712782

[24] Baron, M., Hudson, M., Steele, R. & Canadian Scleroderma Research Group. Malnutrition is common in systemic sclerosis: results from the Canadian scleroderma research group database. *J. Rheumatol.* **36**, 2737–2743 (2009). 10.3899/jrheum.090694 19833750

[25] Marighela TF, Genaro PDS, Pinheiro MM, Szejnfeld VL, Kayser C (2013). Risk factors for body composition abnormalities in systemic sclerosis. Clin. Rheumatol..

[26] Marchand GB (2018). Increased body fat mass explains the positive association between circulating estradiol and insulin resistance in postmenopausal women. Am. J. Physiol. Endocrinol. Metab..

[27] Paccou J, Dewailly J, Cortet B (2012). Reduced levels of serum IGF-1 is related to the presence of osteoporotic fractures in male idiopathic osteoporosis. Joint Bone Spine.

[28] Marot M (2015). Prevalence and predictive factors of osteoporosis in systemic sclerosis patients: a case-control study. Oncotarget.

[29] Hafez EA (2011). Bone mineral density changes in patients with recent-onset rheumatoid arthritis. Clin. Med. Insights Arthritis Musculoskelet. Disord..

[30] Ruaro B (2018). Correlation between bone quality and microvascular damage in systemic sclerosis patients. Rheumatology (Oxford).

[31] Ruaro B (2018). Dickkopf-1 (Dkk-1) serum levels in systemic sclerosis and rheumatoid arthritis patients: correlation with the Trabecular Bone Score (TBS). Clin. Rheumatol..

[32] Neumann K, Wallace DJ, Metzger AL (2000). Osteoporosis–less than expected in patients with scleroderma?. J. Rheumatol..

